# Dietary Chitosan Oligosaccharides Modulate Oxidative Status, Inflammatory and Apoptosis-Related Markers in Immune Organs of Heat-Stressed Broilers

**DOI:** 10.3390/ani16203244

**Published:** 2026-10-15

**Authors:** Haodong Cai, Huan Liu, Yue Wang, Xujia Cao, Xiaoqing Yu, Lilong An, Li Zhang, Tingting Xie

**Affiliations:** 1 College of Coastal Agricultural Sciences, Guangdong Ocean University, Zhanjiang 524088, China; 13025485660@stu.gdou.edu.cn (H.C.); lhuan202610@163.com (H.L.); 2445193354@stu.gdou.edu.cn (Y.W.); 15913150422@stu.gdou.edu.cn (X.C.); yxq328@stu.gdou.edu.cn (X.Y.); anlilong@126.com (L.A.); zhangli761101@163.com (L.Z.); 2 Key Laboratory of Farm Animal Genetic Resources and Germplasm Innovation in Zhanjiang, Guangdong Ocean University, Zhanjiang 524088, China

**Keywords:** chitosan oligosaccharides, heat stress, broilers, inflammatory responses, apoptosis-related gene expression

## Abstract

Chitosan oligosaccharides (COS) have antioxidant and immunomodulatory activities. This study characterized immune-organ responses associated with dietary COS in broilers exposed to cyclic heat stress (HS). A total of 192 birds were assigned to four groups (six cages/group; eight birds/cage): control (CON; 24 ± 1 °C), HS (34 ± 1 °C for 10 h/day), HS + 200 mg/kg COS, and HS + 400 mg/kg COS. Each treatment was replicated in three rooms, with two cages per room. One bird per cage was sampled after the final heat-stress bout and a 12 h fast (*n* = 6/group). Outcomes comprised organ indices and qualitative histology; serum corticosterone; splenic and thymic SOD, CAT, GSH-Px, and MDA; and heat-shock-, cytokine-, and apoptosis-related mRNA. Data were analyzed using a linear mixed model with treatment as a fixed effect and room nested within treatment as a random effect; Tukey-adjusted comparisons followed a significant omnibus test. Treatment was assigned at room level, the CON versus HS contrast was confounded with room temperature condition, and only three rooms were available per treatment, so all inferential comparisons are exploratory. Relative to unsupplemented HS, both COS doses increased spleen and bursa indices (*p* < 0.05), lowered serum corticosterone (*p* < 0.01), increased SOD and CAT activities (*p* < 0.01) and GSH-Px activity (*p* < 0.05), and lowered MDA (*p* < 0.05). COS groups also showed tissue- and dose-dependent shifts in mRNA. The 200 mg/kg group generally showed the largest reductions in pro-inflammatory transcripts, whereas 400 mg/kg produced the most consistent thymic apoptosis-related transcript pattern; no single dose was optimal across all endpoints. Dietary COS was therefore associated with altered immune-organ indices, measured oxidative status, and stress-, cytokine-, and apoptosis-related transcription under HS. Because no thermoneutral + COS group was included and room-level replication was limited, these findings require validation with replicated rooms, protein measurements, and functional immune endpoints.

## Simple Summary

Heat stress can disturb the organs that help chickens develop and coordinate immune responses. We studied 192 broilers in four groups: a normal-temperature control, heat stress without COS, and heat stress with 200 or 400 mg/kg chitosan oligosaccharides (COS). From 28 to 42 days of age, heat-stressed birds were exposed to 34 °C for 10 h/day; one bird from each of six cages per group was sampled. Compared with the unsupplemented heat-stress group, COS-supplemented groups had higher spleen and bursa indices, lower serum corticosterone, higher activities of the measured antioxidant enzymes, lower lipid peroxidation, and altered expression of genes related to heat stress, cytokines, and cell death. However, because the CON versus HS comparison was confounded with room temperature condition and room-level replication was limited, these CON–HS comparisons are exploratory, while the COS-related differences are associative and need confirmation. Therefore, these results are exploratory associations rather than causal evidence for COS. Responses appeared to depend on the organ and dose: a total of 200 mg/kg was generally associated with larger changes in pro-inflammatory transcripts, whereas 400 mg/kg was associated with more consistent changes in thymic apoptosis-related transcripts. These findings do not establish improved immune function because many molecular endpoints were measured only as mRNA and no normal-temperature + COS group was included. The results identify COS-associated patterns that should be tested in room-replicated studies using protein and functional immune measurements.

## 1. Introduction

Global warming and intensive farming practices have exposed livestock production to increasingly severe heat stress (HS) challenges. Heat stress negatively impacts livestock production by reducing growth performance [1], inducing metabolic disorders, decreasing natural antioxidant capabilities [2], and causing immunosuppression [3], leading to substantial economic losses [4]. Given the increasing frequency and severity of extreme heat events under climate change, this economic burden is projected to escalate further [5,6]. Poultry are highly susceptible to heat stress due to their high basal body temperature, feather coverage, and elevated metabolic rate [7]. Heat stress adversely affects poultry health through multiple physiological and molecular pathways [8], seriously restricting the sustainable development of the poultry industry.

The spleen, thymus, and bursa of Fabricius are essential immune organs in chickens. The spleen coordinates systemic immune responses and contains abundant T and B lymphocytes [9], whereas the thymus and bursa of Fabricius support T- and B-cell development, respectively [6,10]. Heat stress has been associated with lower immune-organ weights, disturbed lymphoid architecture, oxidative imbalance, and inflammatory responses [6,11,12]. These changes may interact through an oxidative–inflammatory–apoptotic axis. In broiler spleen, chronic heat exposure has been linked with TLR/MyD88/NF-κB signaling and inflammatory injury [11]. Excess reactive oxygen species can exceed antioxidant defenses, interact with NF-κB and MAPK signaling, damage mitochondria, and alter *cytochrome c-*, *caspase-*, and *BCL-2*-family pathways [13,14,15,16,17,18,19,20]. These pathways provide a plausible link among redox imbalance, inflammatory transcription, and mitochondrial apoptosis; however, transcript abundance alone cannot establish pathway activation or apoptosis. Identifying nutritional approaches that modify these responses may help define strategies for broilers exposed to high ambient temperatures.

Chitosan oligosaccharides (COS) are glucosamine-rich oligosaccharides derived from chitin or chitosan and are valued for their solubility, biocompatibility, and low toxicity [21,22]. Poultry studies have associated dietary COS with growth, antioxidant, intestinal, and immune-organ outcomes [23,24]. Proposed mechanisms are context dependent and include ERK1/2-mediated enhancement of HO-1 and GSH-Px expression [23], reduced NF-κB nuclear translocation [25], CaSR–PLCβ2/AMPK signaling [26], and maintenance of intestinal barrier integrity [27]. Much of this evidence derives from muscle, intestinal, or non-poultry models and therefore cannot be assumed to operate in immune organs. Previous poultry work has established effects on performance, intestinal health, oxidative markers, and immune-organ indices, but tissue- and dose-resolved transcriptional responses in the spleen, thymus, and bursa of Fabricius during cyclic HS remain insufficiently defined. We therefore evaluated organ indices and histology, measured oxidative status, and stress-, cytokine-, and apoptosis-related transcripts in these organs. We hypothesized that dietary COS would attenuate HS-associated oxidative and inflammatory alterations and modify apoptosis-related transcription in a tissue- and dose-dependent manner.

## 2. Materials and Methods

### 2.1. Ethics Statement

All animal experiments were approved by the Animal Care and Use Committee of Guangdong Ocean University (Approval Number: 20240007). All experimental procedures were conducted in accordance with the regulations and guidelines established by the committee. The principles of the 3Rs (Replacement, Reduction, and Refinement) were strictly followed throughout this study. At the conclusion of the experiment, all broilers were humanely euthanized by cervical dislocation following a 12 h fasting period, and all procedures were performed to minimize animal suffering.

### 2.2. Animals and Experimental Design

A total of 300 one-day-old male Arbor Acres (AA) broiler chicks were obtained from Zhengda Broiler Hatchery (Zhanjiang, China). All broilers were provided with unrestricted access to feed and water and were fed the same basal diet until they reached 28 days of age. On day 28, 192 healthy broilers with similar body weights were allocated, after stratification by initial body weight, to four groups (six cages/group; eight birds/cage): (1) CON, basal diet at thermoneutral temperature; (2) HS, basal diet under cyclic heat stress; (3) HS + 200 mg/kg COS; and (4) HS + 400 mg/kg COS. During days 28–42, CON rooms were maintained at 24 ± 1 °C, whereas HS rooms were maintained at 34 ± 1 °C from 08:00 to 18:00 and at 24 ± 1 °C for the remaining 14 h; relative humidity was maintained at 65 ± 5%. All rooms used the same environmental-control and management procedures. The lighting schedule was 16 h light:8 h dark at 5–10 lux, and ventilation, airflow, feeding schedule, dietary composition, and water availability were standardized. A three-tier cage system (124 × 64 × 40 cm) was used; eight birds occupied each 0.794 m^2^ cage (approximately 10.1 birds/m^2^), and cage positions were distributed to reduce location effects. Temperature and relative humidity were monitored continuously by the room environmental-control system.

Each treatment was assigned to three rooms, with two cages per room and eight birds per cage (six cages per treatment). Thus, the experiment used 12 rooms in total: 3 rooms for CON, 3 rooms for HS, 3 rooms for HS + 200 mg/kg COS, and 3 rooms for HS + 400 mg/kg COS. Rooms were nested within treatment. Because treatment was assigned at the room level, room-level factors could not be fully separated from treatment; in particular, the CON versus HS comparison was confounded with room temperature condition. Because only three rooms were used per treatment, room-level variance was estimated with limited precision. One bird was randomly selected from each cage for blood and tissue collection, and the sampled bird represented its cage for bird-level endpoints (nominal *n* = 6/group).

Chitosan oligosaccharides (COS, purity: 93.6%, average molecular weight 1768 Da, degree of deacetylation ≥ 90%) were purchased from Qingdao Songtian Biotechnology Co., Ltd. (Qingdao, China). The basal diet was formulated according to the Chinese Broiler Feeding Standard (NY/T 33-2004) [28]. The specific composition and nutrient levels of the basal diet are detailed in Supplementary Table S1. Previously conducted dose-gradient exploratory work had been done. In the preliminary experiment, we evaluated the effects of different COS levels on performance and mortality, and the COS supplementation level finally selected for the formal experiment had no significant effect on growth performance compared with the normal control group and was associated with a mortality rate below 5%. In addition, based on the aims of the present study and previous literature, two doses of 200 and 400 mg/kg were selected [23,24]. COS was first premixed with a small quantity of basal feed and then blended into the complete batch. No thermoneutral + COS group was included, so independent COS effects cannot be estimated.

### 2.3. Sample Collection

On day 42, after the final heat-stress bout had ended at 18:00 on the preceding cycle, birds underwent a 12 h fasting period and were sampled at 06:00, exactly 12 h after the end of the final heat-stress exposure. One broiler was randomly selected from each replicate cage (*n* = 6 per treatment) and weighed. Blood samples were collected from the wing vein using sterile syringes, transferred into centrifuge tubes, and centrifuged at 3000× *g* for 15 min at 4 °C to obtain serum, which was aliquoted and stored at −80 °C. After blood collection, broilers were humanely euthanized by cervical dislocation. The thymus, spleen, and bursa of Fabricius were rapidly excised, cleared of adherent tissue, weighed for organ index calculation, snap-frozen in liquid nitrogen, and stored at −80 °C for subsequent analyses of antioxidant status and gene expression profiles. Additionally, 1 cm^3^ of each tissue was fixed in 4% paraformaldehyde (Beyotime Biotechnology Co., Ltd., Shanghai, China, P0099) for histopathological analysis.

### 2.4. Immune-Organ Growth Index Determination

Immune-organ index (%) = [Immune-organ weight (g)/Live body weight (g)] × 100

### 2.5. Histological Examination

Spleen and thymus samples were collected from standardized anatomical sites: the mid-portion of the spleen and cranial cervical thymic lobes. Fixed tissues were paraffin embedded, sectioned, stained with hematoxylin and eosin (Beyotime Biotechnology Co., Ltd., Shanghai, China, C0105S), and examined by light microscopy(Nikon, Tokyo, Japan). For each sampled bird, three sections per organ and three non-overlapping fields per section were photographed at 20× magnification. The examination was descriptive; the investigator was not blinded to treatment, and no validated lesion score, morphometric outcome, or blinded inferential comparison was performed. Histology was limited to the spleen and thymus, so bursal interpretation was restricted to the organ index and transcript measurements.

### 2.6. Biochemical Assays

Serum corticosterone (H205-1), SOD (A001-3), CAT (A007-2), GSH-Px (A005-1), and MDA (A003) were measured in spleen and thymus homogenates with commercial kits from Nanjing Jiancheng Bioengineering Institute (Nanjing, China) following the manufacturers’ procedures. Absorbance was read with a DNM-9606 microplate reader (Beijing Puran Biotechnology Co., Ltd., Beijing, China). SOD activity and MDA content were normalized to wet tissue (U/g tissue and nmol/g tissue, respectively), CAT activity was normalized to protein (μmol/min/mg protein), and GSH-Px activity was normalized to homogenate volume (nmol/min/mL). Antioxidant assays were not performed in the bursa of Fabricius, and no inference about bursal redox status was made.

### 2.7. RNA Extraction, Reverse Transcription, and Quantitative Real-Time PCR (qRT-PCR)

Frozen spleen, thymus, and bursa of Fabricius samples were homogenized in 1 mL TRIzol reagent and total RNA extraction kit(Meiji Biotechnology Co., Ltd., Guangzhou, China), and total RNA was extracted according to the manufacturer’s instructions. RNA concentration and purity were assessed spectrophotometrically; samples with OD260/OD280 ratios of 1.9–2.0 were used. Reverse transcription was performed with a commercial kit (Nanjing Vazyme Biotechnology Co., Ltd., Nanjing, China, AU341). qRT-PCR used SYBR Green Premix Pro Taq HS reagent (Nanjing Vazyme Biotechnology Co., Ltd., Nanjing, China, Q711) on a CFX96 Real-Time PCR Detection System (Bio-Rad, Hercules, CA, USA), with *β-actin* as the reference gene. The cycling program was 95 °C for 30 s, followed by 40 cycles of 95 °C for 10 s, 58 °C for 30 s, and 72 °C for 15 s. In addition, the data used for relative expression analysis met the following criteria: standard-curve slope between −3.5 and −3.4, R^2^ > 0.98, Ct < 33 for 10-fold dilutions, standard deviation of replicate Ct values < 0.5, and a single peak in the melt curve. Relative mRNA abundance was calculated by the 2^−ΔΔCt^ method; primer sequences are listed in Table 1. The primers were synthesized by Sangon Biotech Co., Ltd., Shanghai, China.

### 2.8. Statistical Analysis

Data are presented as mean ± SEM for six sampled cages per group. Because each treatment was assigned to three rooms and each room contained two cages, the nominal model for each endpoint was a linear mixed model: Yij = μ + Ti + Rj(i) + εij, where Ti was the fixed effect of treatment, Rj(i) was the random effect of room nested within treatment, and εij was the residual variation among cage-level observations; one bird per cage represented the cage for bird-level endpoints (*n* = 6 cages/group). Treatment was assigned at the room level, so room-level factors could not be fully separated from treatment, and the CON versus HS contrast was additionally confounded with room temperature condition. Because only three rooms were used per treatment, room-level variance was estimated with limited precision, and all inferential comparisons are exploratory. When the omnibus test was significant, Tukey-adjusted pairwise comparisons of estimated marginal means were performed. Tukey adjustment controlled multiplicity within each endpoint; no global false-discovery-rate correction was applied across the many tissues and transcripts, so the molecular findings are exploratory. Formal linear or quadratic COS contrasts were not performed because only two COS doses were available within the HS treatments; apparent dose patterns are described rather than treated as established trends. All *p* values and CON versus HS or dietary contrasts are accordingly interpreted as exploratory associations. Analyses used SPSS 25.0 (IBM Corp., Armonk, NY, USA), and figures were prepared in GraphPad Prism 10.4.2 (GraphPad Software, San Diego, CA, USA); *p* < 0.05 was the nominal significance threshold.

## 3. Results

### 3.1. Immune-Organ Responses to COS Supplementation Under Cyclic Heat Stress

**Figure 1:**
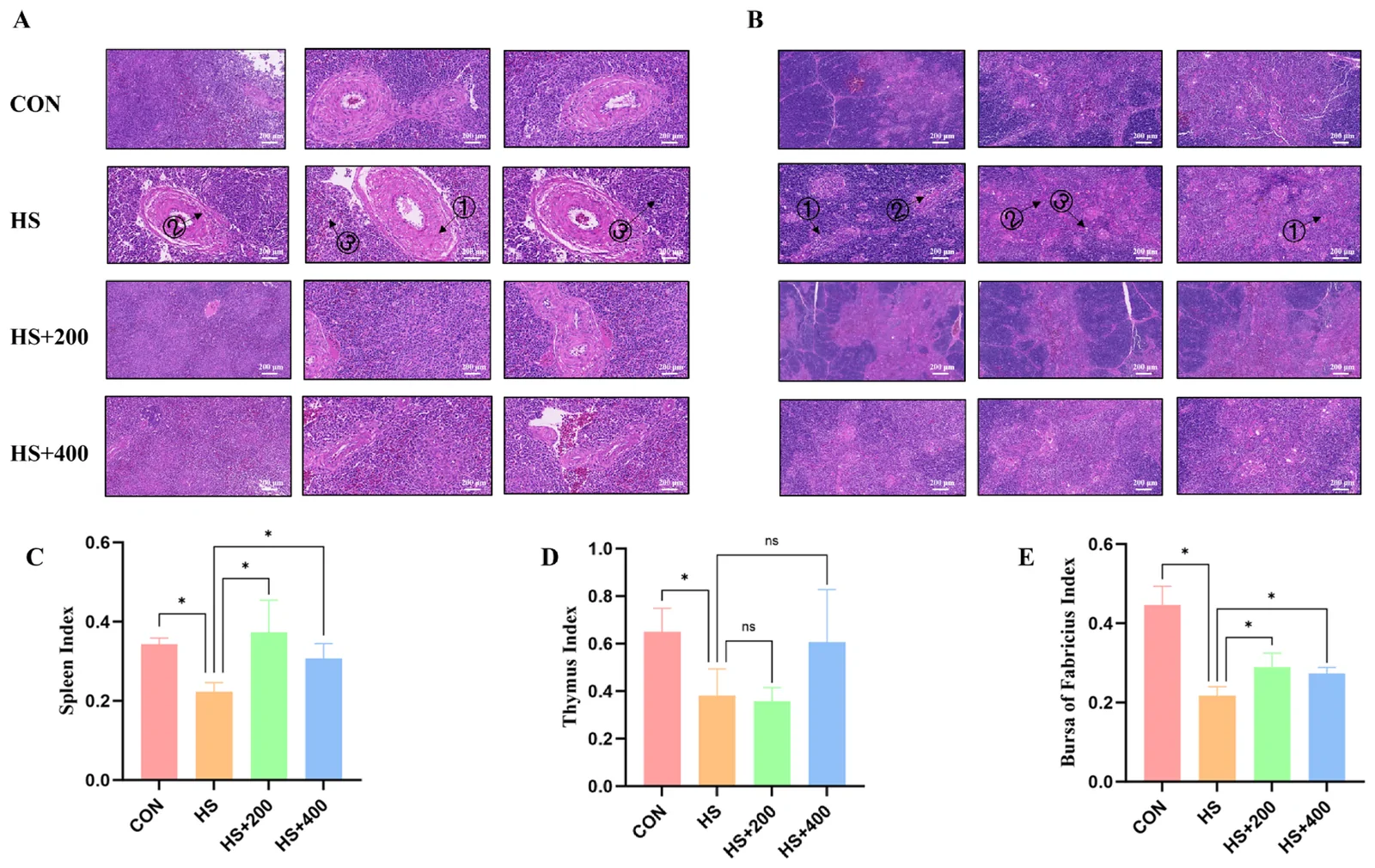
Immune-organ histology and indices across dietary COS treatments under cyclic heat stress. (**A**,**B**) Representative H&E-stained spleen and thymus sections; arrows identify central-artery wall thickening/luminal narrowing (1), erythrocyte extravasation (2), and cell necrosis (3) in the displayed fields. These panels are qualitative and were not subjected to lesion scoring or morphometric statistics. (**C**–**E**) Spleen, thymus, and bursa of Fabricius indices (mean ± SEM; *n* = 6 sampled cages/group). Brackets show the prespecified Tukey-adjusted pairwise comparisons of CON, HS + 200, and HS + 400 with HS in the source graphs: * *p* < 0.05 and ns, *p* > 0.05. COS, chitosan oligosaccharides; CON, 24 ± 1 °C control; HS, 34 ± 1 °C cyclic heat stress; HS + 200 and HS + 400, HS with 200 or 400 mg/kg COS, respectively.

Representative spleen and thymus sections are shown in Figure 1A,B. In the displayed HS sections, splenic central-artery wall thickening and luminal narrowing and thymic erythrocyte extravasation, hemorrhage, and cell necrosis were visible. The displayed COS sections appeared qualitatively closer to the CON sections. Because histology was not evaluated with blinded scoring or morphometry, these images are descriptive and were not used for statistical between-group inference. Quantitatively, the HS group exhibited lower spleen, thymus, and bursa indices than CON (*p* < 0.05; Figure 1C–E). Relative to HS, both COS groups had higher spleen and bursa indices (*p* < 0.05), whereas the thymus index did not differ at either COS dose (Figure 1C–E).

### 3.2. HSP Transcript and Serum Corticosterone Responses to COS Under Cyclic Heat Stress

**Figure 2:**
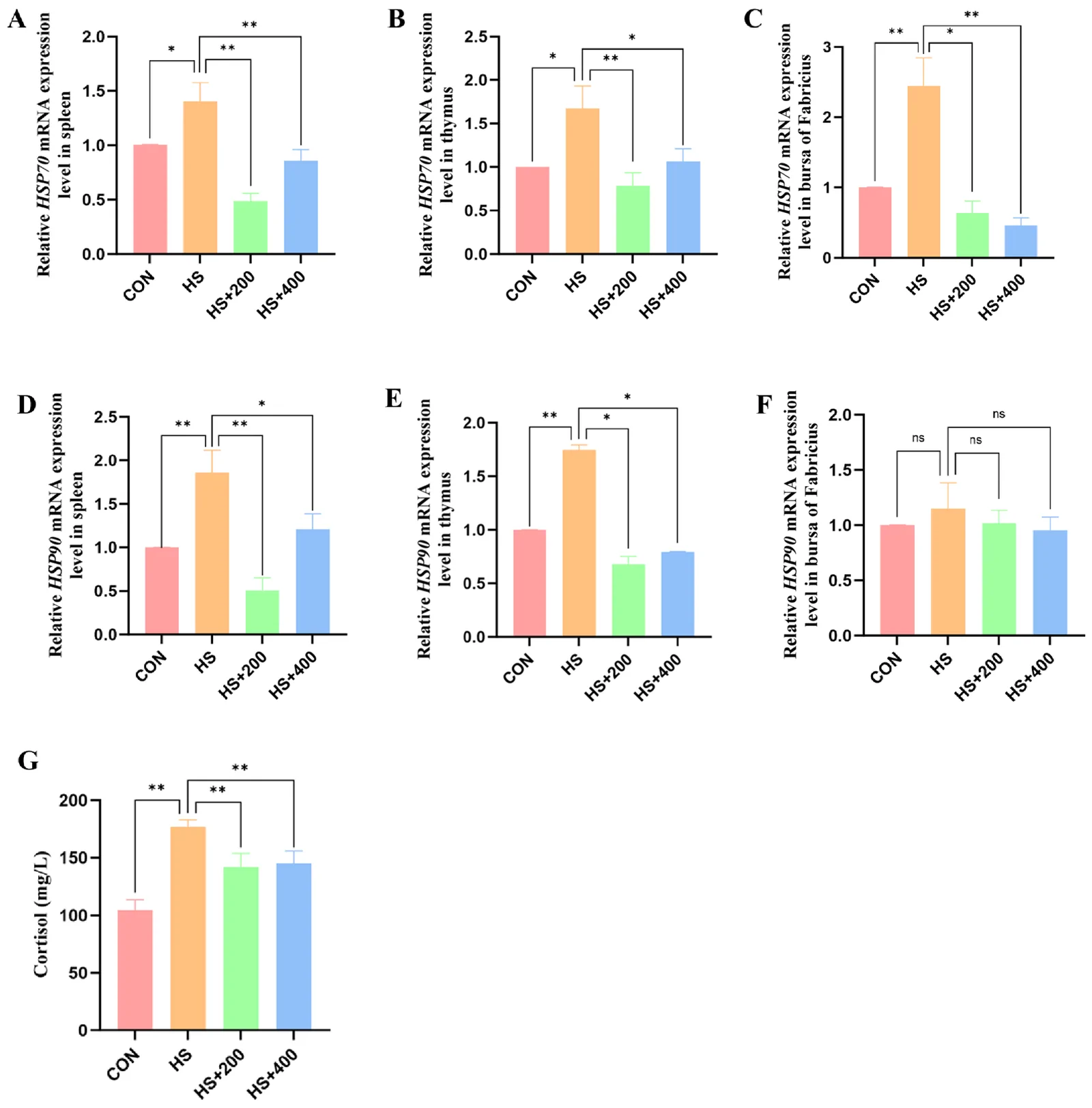
*HSP70* and *HSP90* transcript abundance and serum corticosterone concentrations across treatment groups. (**A**–**C**) Relative mRNA expression levels of *HSP70* in the spleen (**A**), thymus (**B**), and bursa of Fabricius (**C**). (**D**–**F**) Relative mRNA expression levels of *HSP90* in the spleen (**D**), thymus (**E**), and bursa of Fabricius (**F**). (**G**) Serum corticosterone concentrations. Data are presented as mean ± SEM (*n* = 6 per group). The CON, HS + 200, and HS + 400 groups were each compared with the HS group. * *p* < 0.05, ** *p* < 0.01, and ns, *p* > 0.05. COS, chitosan oligosaccharides; CON, normal temperature treatment (24 ± 1 °C), control group; HS, high temperature treatment (34 ± 1 °C), heat-stress group; HS + 200, heat-stressed broilers supplemented with 200 mg/kg COS; HS + 400, heat-stressed broilers supplemented with 400 mg/kg COS.

The *HSP70* and *HSP90* mRNA results and serum corticosterone concentrations are shown in Figure 2. Relative to CON, the HS group exhibited higher *HSP70* mRNA abundance in the spleen and thymus (*p* < 0.05) and bursa of Fabricius (*p* < 0.01), higher *HSP90* mRNA abundance in the spleen and thymus (*p* < 0.01), and higher serum corticosterone (*p* < 0.01); bursal *HSP90* did not differ (*p* > 0.05). Relative to HS, both COS groups had lower *HSP70* mRNA abundance in all three organs and lower *HSP90* mRNA abundance in spleen and thymus (*p* < 0.05), while bursal *HSP90* did not differ. Both COS groups had lower serum corticosterone (*p* < 0.01).

### 3.3. Cytokine-Transcript Responses to COS Under Cyclic Heat Stress

**Figure 3:**
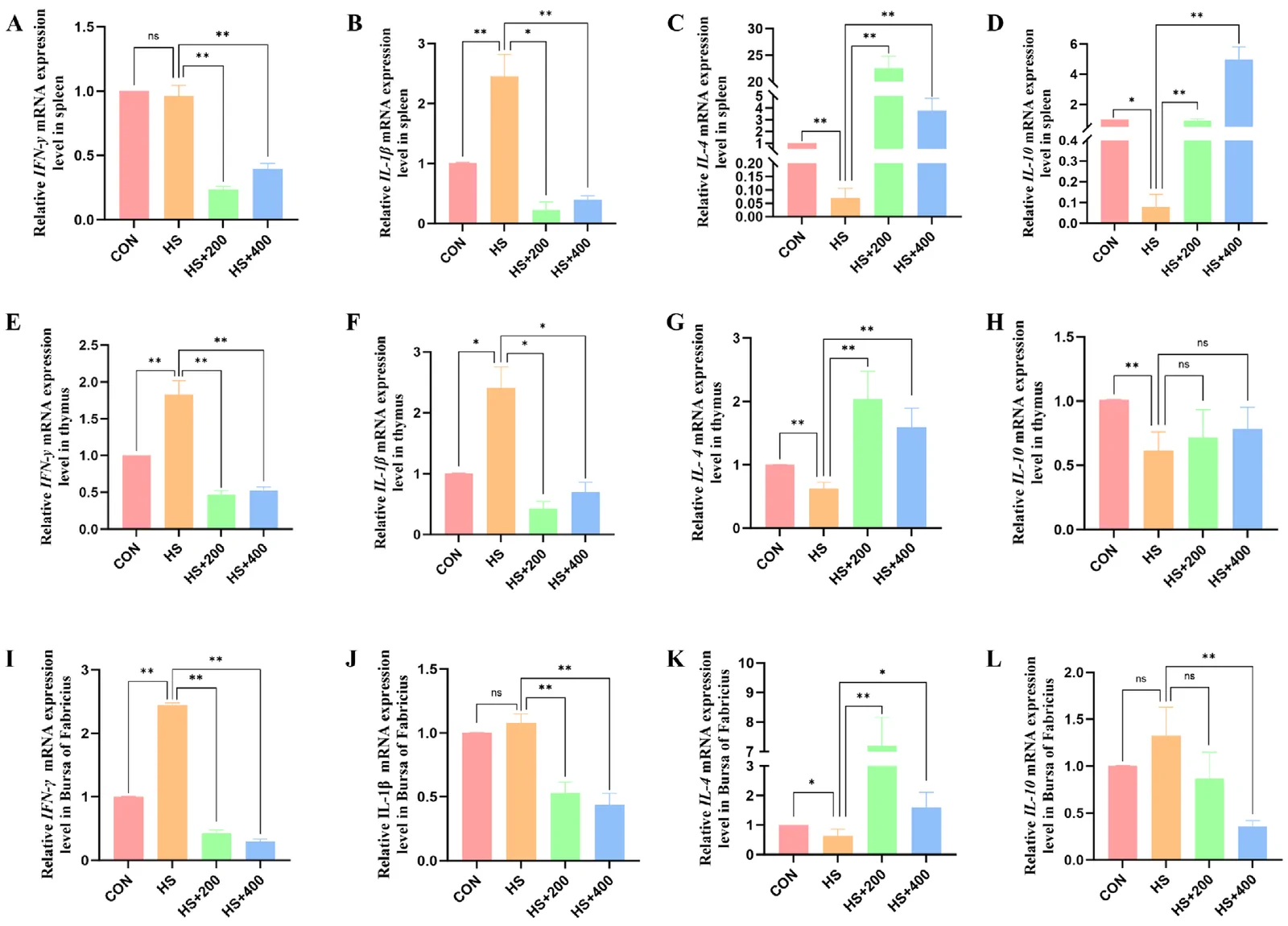
Cytokine-related transcript abundance in the spleen, thymus, and bursa of Fabricius of broilers under cyclic heat stress. (**A**–**D**): Relative mRNA expression levels of *IFN-γ* (**A**), *IL-1β* (**B**), *IL-4* (**C**), and *IL-10* (**D**) in spleen; (**E**–**H**): Relative mRNA expression levels of IFN-γ (**E**), *IL-1β* (**F**), *IL-4* (**G**), and *IL-10* (**H**) in thymus; (**I**–**L**): Relative mRNA expression levels of *IFN-γ* (**I**), *IL-1β* (**J**), *IL-4* (**K**), and *IL-10* (**L**) in bursa of Fabricius. Data are presented as mean ± SEM (*n* = 6 per group). The CON, HS + 200, and HS + 400 groups were each compared with the HS group. * *p* < 0.05, ** *p* < 0.01, and ns, *p* > 0.05. COS, chitosan oligosaccharides; CON, normal temperature treatment (24 ± 1 °C), control group; HS, high temperature treatment (34 ± 1 °C), heat-stress group; HS + 200, heat-stressed broilers supplemented with 200 mg/kg COS; HS + 400, heat-stressed broilers supplemented with 400 mg/kg COS.

Cytokine-related mRNA abundance is shown in Figure 3. Relative to CON, the HS group exhibited higher *IL-1β* and *IFN-γ* mRNA in most tissues, except splenic *IFN-γ* (*p* > 0.05), and lower *IL-4* mRNA in all three organs and *IL-10* mRNA in spleen and thymus (*p* < 0.05); bursal *IL-10* did not differ. Relative to HS, both COS groups had lower *IFN-γ* and *IL-1β* mRNA and higher *IL-4* and *IL-10* mRNA in the measured organs, with generally larger changes in pro-inflammatory transcripts at 200 mg/kg. These measurements indicate a transcriptional pattern and should not be interpreted as cytokine protein concentrations or a direct functional measure of inflammation.

### 3.4. Oxidative-Status Responses to COS Under Cyclic Heat Stress

**Figure 4:**
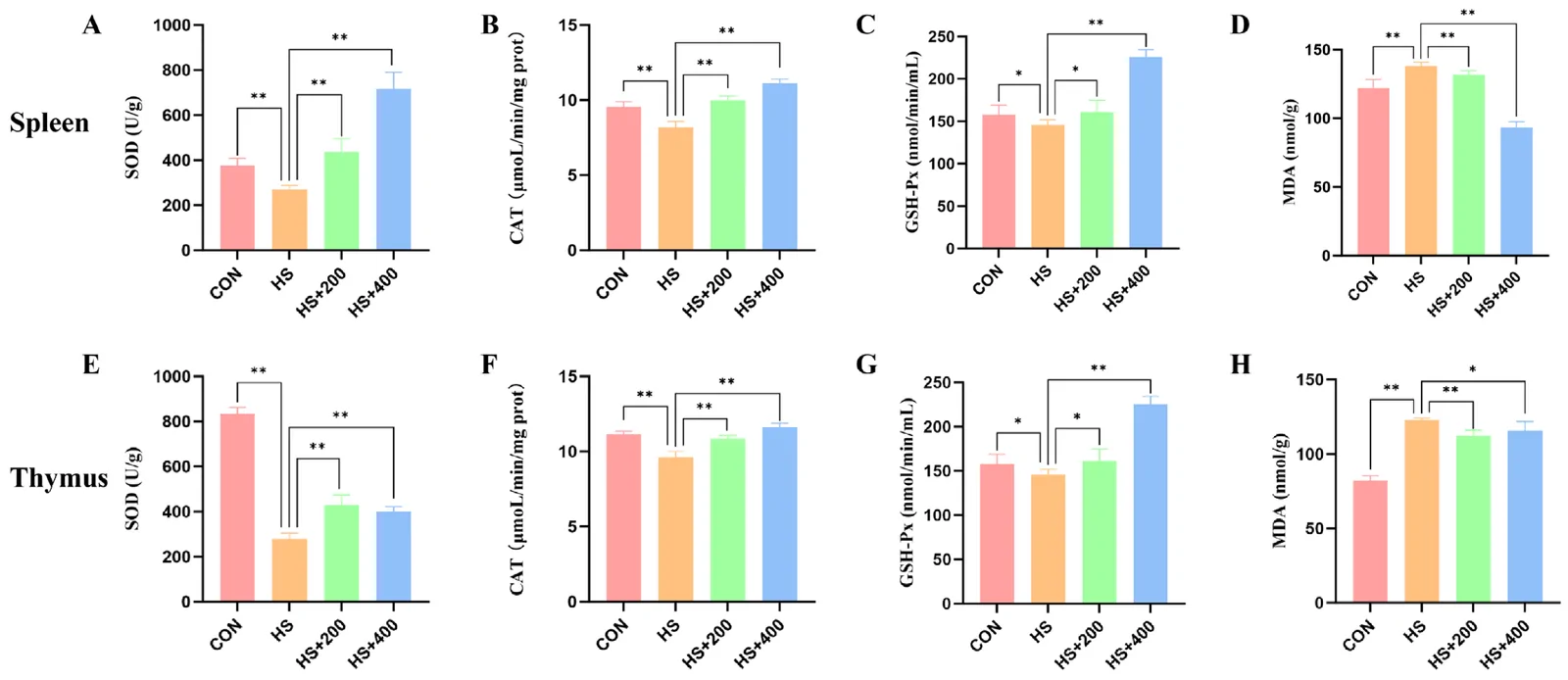
Oxidative-status markers in the spleen and thymus of broilers under cyclic heat stress. (**A**–**D**): SOD (**A**), CAT (**B**), and GSH-Px (**C**) activities and MDA (**D**) content in spleen; (**E**–**H**): SOD (**E**), CAT (**F**), and GSH-Px (**G**) activities and MDA (**H**) content in thymus. Data are presented as mean ± SEM (*n* = 6 per group). The CON, HS + 200, and HS + 400 groups were each compared with the HS group. * *p* < 0.05, ** *p* < 0.01. COS, chitosan oligosaccharides; CON, normal temperature treatment (24 ± 1 °C), control group; HS, high temperature treatment (34 ± 1 °C), heat-stress group; HS + 200, heat-stressed broilers supplemented with 200 mg/kg COS; HS + 400, heat-stressed broilers supplemented with 400 mg/kg COS.

To characterize measured oxidative status, SOD, CAT, and GSH-*P*x activities and MDA content were assessed in the spleen and thymus (Figure 4). Relative to CON, the HS group had lower SOD and CAT activities (*p* < 0.01), lower GSH-*P*x activity (*p* < 0.05), and higher MDA (*p* < 0.01). Relative to HS, both COS groups had higher SOD and CAT activities (*p* < 0.01), higher GSH-*P*x activity (*p* < 0.05), and lower MDA (*p* < 0.05) in both tissues. These endpoints support differences in the measured antioxidant/oxidative status but do not characterize the entire cellular redox system.

### 3.5. Apoptosis-Related Transcript Responses to COS Under Cyclic Heat Stress

**Figure 5:**
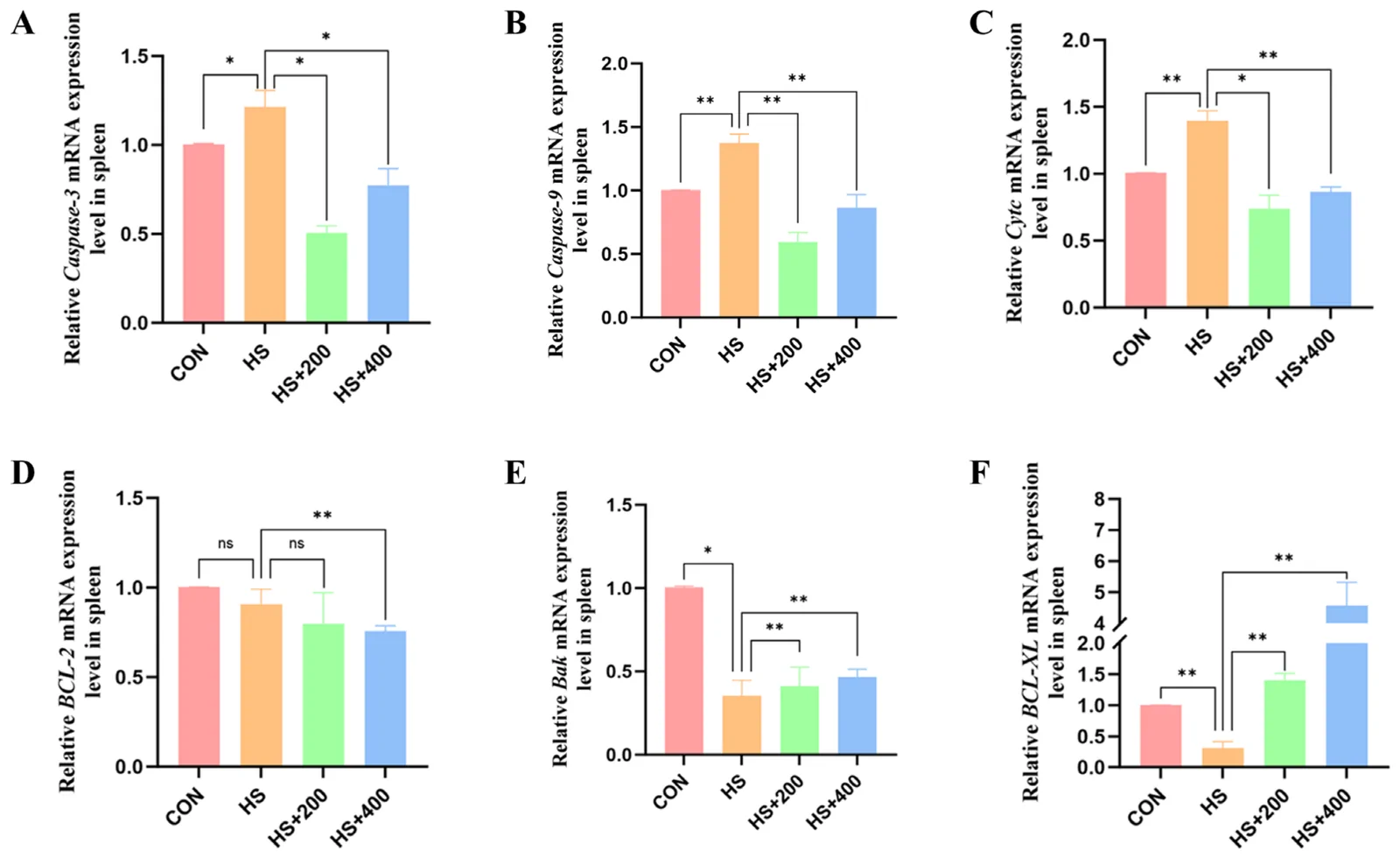
Apoptosis-related gene expression in the spleen of broilers under cyclic heat stress. (**A**–**F**): Relative mRNA expression levels of *Caspase-3* (**A**), *Caspase-9* (**B**), *Cyt c* (**C**), *BCL-2* (**D**), *Bak* (**E**), and *BCL-XL* (**F**) in spleen. Data are presented as mean ± SEM (*n* = 6 per group). The CON, HS + 200, and HS + 400 groups were each compared with the HS group. * *p* < 0.05, ** *p* < 0.01, and ns, *p* > 0.05. COS, chitosan oligosaccharides; CON, normal temperature treatment (24 ± 1 °C), control group; HS, high temperature treatment (34 ± 1 °C), heat-stress group; HS + 200, heat-stressed broilers supplemented with 200 mg/kg COS; HS + 400, heat-stressed broilers supplemented with 400 mg/kg COS.

**Figure 6:**
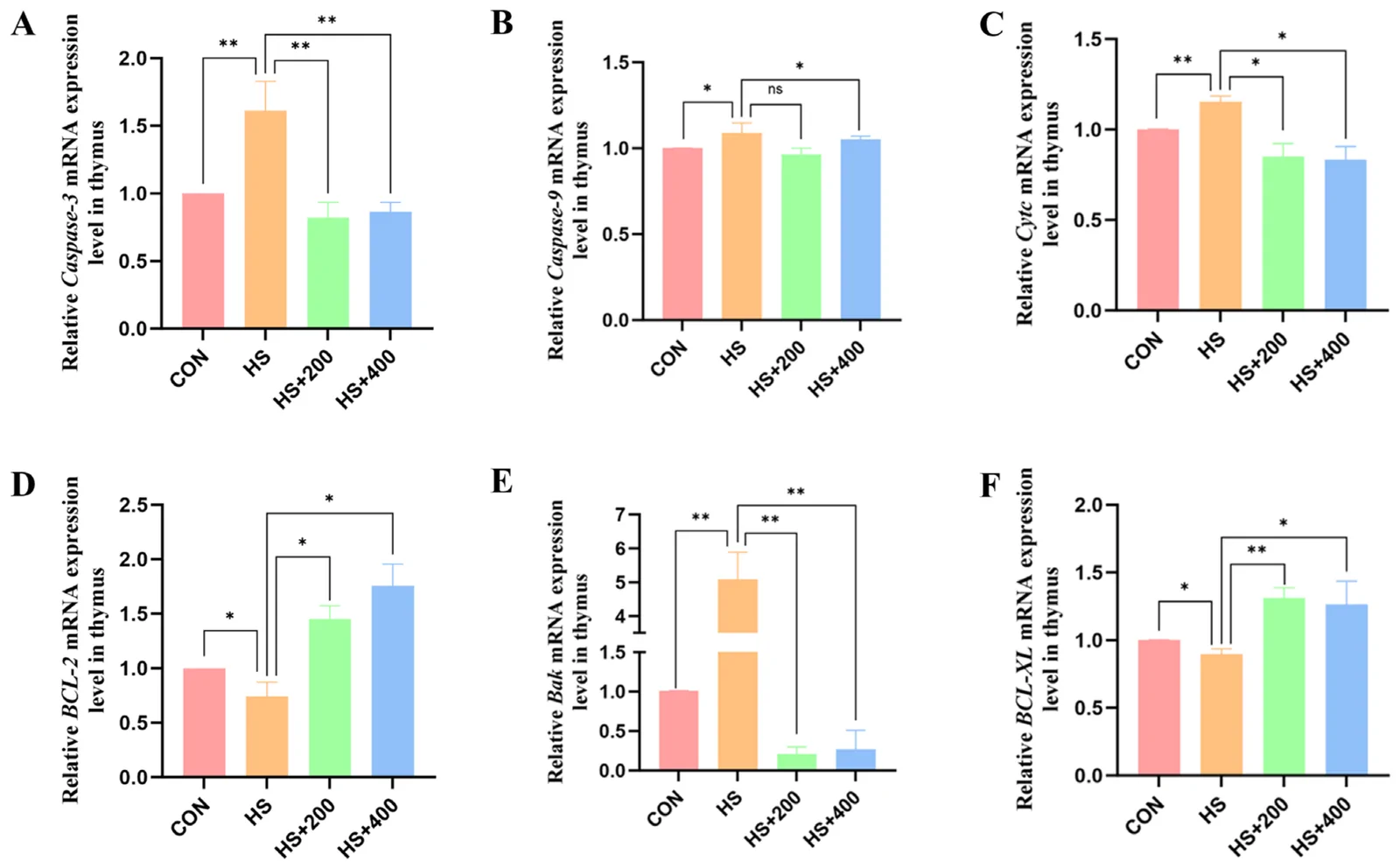
Apoptosis-related gene expression in the thymus of broilers under cyclic heat stress. (**A**–**F**): Relative mRNA expression levels of *Caspase-3* (**A**), *Caspase-9* (**B**), *Cyt c* (**C**), *BCL-2* (**D**), *Bak* (**E**), and *BCL-XL* (**F**) in thymus. Data are presented as mean ± SEM (*n* = 6 per group). The CON, HS + 200, and HS + 400 groups were each compared with the HS group. * *p* < 0.05, ** *p* < 0.01, and ns, *p* > 0.05. COS, chitosan oligosaccharides; CON, normal temperature treatment (24 ± 1 °C), control group; HS, high temperature treatment (34 ± 1 °C), heat-stress group; HS + 200, heat-stressed broilers supplemented with 200 mg/kg COS; HS + 400, heat-stressed broilers supplemented with 400 mg/kg COS.

**Figure 7:**
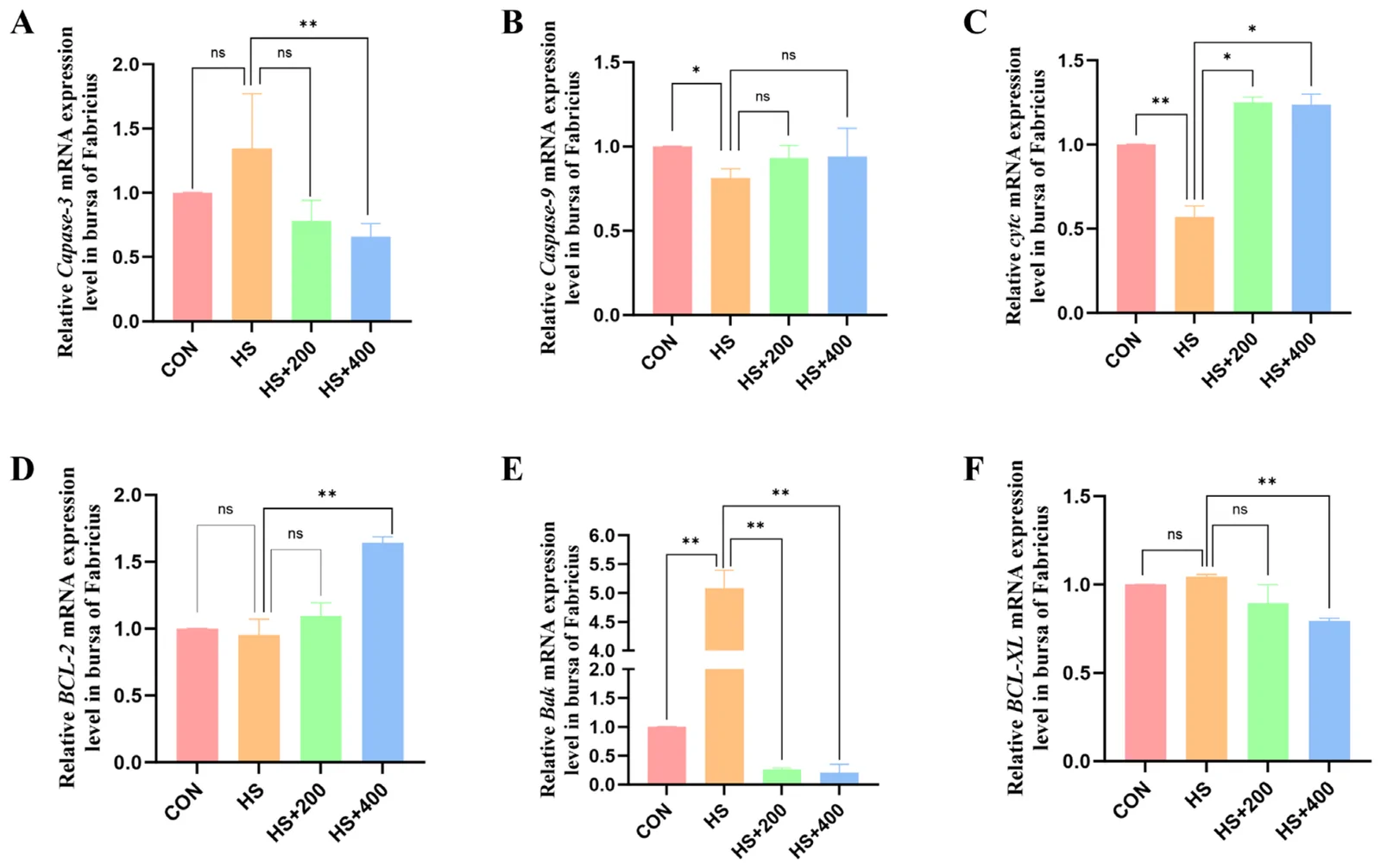
Apoptosis-related gene expression in the bursa of Fabricius of broilers under cyclic heat stress. (**A**–**F**): Relative mRNA expression levels of *Caspase-3* (**A**), *Caspase-9* (**B**), *Cyt c* (**C**), *BCL-2* (**D**), *Bak* (**E**), and *BCL-XL* (**F**) in bursa of Fabricius. Data are presented as mean ± SEM (*n* = 6 per group). The CON, HS + 200, and HS + 400 groups were each compared with the HS group. * *p* < 0.05, ** *p* < 0.01, and ns, *p* > 0.05. COS, chitosan oligosaccharides; CON, normal temperature treatment (24 ± 1 °C), control group; HS, high temperature treatment (34 ± 1 °C), heat-stress group; HS + 200, heat-stressed broilers supplemented with 200 mg/kg COS; HS + 400, heat-stressed broilers supplemented with 400 mg/kg COS.

Apoptosis-related mRNA abundance in the spleen, thymus, and bursa of Fabricius is shown in Figure 5, Figure 6 and Figure 7.

In the spleen (Figure 5), the HS group had higher *Caspase-3*, *Caspase-9*, and *Cyt c* mRNA expression levels than the CON group (*p* < 0.05; Figure 5A–C), together with lower *BCL-XL* (*p* < 0.01; Figure 5F) and *Bak* (*p* < 0.05) mRNA expression levels. *BCL-2* did not differ between the CON and HS groups (*p* > 0.05; Figure 5D). Within the HS treatments, 200 mg/kg COS was associated with lower *Caspase-3* and *Cyt c* (*p* < 0.05) and *Caspase-9* (*p* < 0.01) mRNA expression levels; the 400 mg/kg COS treatment was associated with lower *Caspase-3* (*p* < 0.05), *Caspase-9* (*p* < 0.01), and *Cyt c* (*p* < 0.01) mRNA expression levels. Both COS doses were associated with higher *Bak* and *BCL-XL* mRNA expression levels (*p* < 0.01). *BCL-2* did not differ at 200 mg/kg COS (*p* > 0.05) but was lower at 400 mg/kg COS (*p* < 0.01).

In the thymus (Figure 6), the HS group had higher *Caspase-3* and *Cyt c* (both *p* < 0.01), *Caspase-9* (*p* < 0.05), and *Bak* (*p* < 0.01) mRNA expression levels and lower *BCL-2* and *BCL-XL* mRNA expression levels (both *p* < 0.05) than the CON group (Figure 6A–F). Within the HS treatments, 200 mg/kg COS was associated with lower *Caspase-3* (*p* < 0.01), *Cyt c* (*p* < 0.05), and *Bak* (*p* < 0.01) mRNA expression levels, whereas *Caspase-9* did not differ (*p* > 0.05). At 400 mg/kg COS, the expression levels of *Caspase-3* and *Bak* were lower (both *p* < 0.01), and those of *Caspase-9* and *Cyt c* were lower (both *p* < 0.05), than in the unsupplemented HS group. Both COS doses were associated with higher *BCL-2* mRNA expression levels (*p* < 0.05); *BCL-XL* expression was also higher at 200 and 400 mg/kg COS (*p* < 0.01 and *p* < 0.05, respectively).

In the bursa of Fabricius (Figure 7), the HS group had higher *Bak* mRNA expression (*p* < 0.01) but lower *Caspase-9* (*p* < 0.05) and *Cyt c* (*p* < 0.01) mRNA expression than the CON group. *Caspase-3*, *BCL-2*, and *BCL-XL* did not differ (*p* > 0.05). Within the HS treatments, 200 mg/kg COS was associated with higher *Cyt c* (*p* < 0.05) and lower *Bak* (*p* < 0.01) mRNA expression, while *Caspase-3*, *Caspase-9*, *BCL-2*, and *BCL-XL* did not differ (*p* > 0.05). At 400 mg/kg COS, the mRNA expression of *Caspase-3* and *Bak* was lower (both *p* < 0.01), that of *Cyt c* and *BCL-2* was higher (*p* < 0.05 and *p* < 0.01, respectively), and *BCL-XL* mRNA expression was lower (*p* < 0.01); *Caspase-9* did not differ (*p* > 0.05).

## 4. Discussion

Heat stress is an important constraint on poultry production and is associated with neuroendocrine, oxidative, inflammatory, and immune-organ alterations [29,30]. The spleen, thymus, and bursa of Fabricius serve different immune functions [8,9,31], which provides a biological basis for organ-specific responses. In this study, the HS group differed from CON in immune-organ indices and architecture, serum corticosterone, measured oxidative status, and stress-, cytokine-, and apoptosis-related transcripts. Because treatment was assigned at the room level and room-level factors could not be fully separated from treatment, these contrasts are exploratory. Within the nominal HS comparisons, COS groups showed partial shifts in these endpoints toward the CON pattern, but the data demonstrate associations, not causal pathway activation or improved functional immunity.

The HS group had higher *HSP70* and *HSP90* mRNA abundance in spleen and thymus, higher bursal *HSP70* mRNA, and higher serum corticosterone than CON. *HSP70* and *HSP90* are inducible molecular chaperones that support protein folding and cellular homeostasis during stress [32,33], while elevated corticosterone is consistent with systemic stress activation [34]. Lower HS*P* transcripts and corticosterone in the COS groups may reflect reduced activation of these stress responses, consistent with prior COS studies [35]. Nevertheless, HS*P*s are also adaptive and cytoprotective; lower transcript abundance is not direct evidence of improved cellular protection, and the absence of HS*P* protein measurements precludes a stronger conclusion.

Relative to the CON group, the HS group showed a predominantly pro-inflammatory transcriptional profile in the immune organs. Except for splenic *IFN-γ* (which was not elevated), the mRNA expression levels of *IL-1β* and *IFN-γ* were higher in most tissues, whereas *IL-4* mRNA expression levels were lower in the spleen, thymus, and bursa of Fabricius, and *IL-10* mRNA expression levels were lower in the spleen and thymus. Bursal *IL-10* did not differ. This pattern is consistent with an altered balance between pro- and anti-inflammatory transcripts in the HS treatment setting, but the room-level design precludes attributing the differences solely to temperature. The tissue-specific patterns may relate to differences in resident immune-cell populations, local cytokine networks [36,37], and the functions of the spleen, thymus, and bursa of Fabricius [38,39].

Within the nominal HS comparisons, COS groups generally had lower *IL-1β* and *IFN-γ* mRNA and higher *IL-4* and *IL-10* mRNA. This pattern is compatible with, but does not prove, a less pro-inflammatory state [35,40]. Several pro-inflammatory transcripts changed more at 200 than at 400 mg/kg, indicating an apparently non-linear response. Saturation of uptake, differences in bioavailability, hormetic responses, or feedback regulation are possible explanations; these findings require further validation. NF-κB-related signaling is another mechanism reported in other COS models [25]; it remains hypothetical in the present organs because NF-κB activation, upstream regulators, and cytokine proteins were not measured.

Relative to CON, the HS group had lower SOD, CAT, and GSH-*P*x activities and higher MDA in spleen and thymus, while both COS groups had higher enzyme activities and lower MDA than HS. This pattern is consistent with improved status for the measured antioxidant and lipid-peroxidation endpoints and agrees with prior poultry heat-stress work [41,42]. It should not be equated with complete resolution of oxidative stress because reactive oxygen species, total antioxidant capacity, GSH/GSSG, protein oxidation, and mitochondrial function were not measured and the bursa was not assayed.

In the spleen, the apoptosis-related transcriptional pattern was not uniformly consistent with a typical mitochondrial apoptotic response. Relative to the CON group, the HS group had higher *Caspase-3*, *Caspase-9*, and *Cyt c* mRNA abundance, lower *Bak* and *BCL-XL* mRNA abundance, and unchanged *BCL-2* mRNA abundance. Within the HS treatments, COS supplementation was associated with lower *Caspase-3* and *Caspase-9* and higher *BCL-XL* mRNA abundance but also with higher *Bak* mRNA abundance; at 400 mg/kg COS, *BCL-2* mRNA abundance was lower. These divergent changes support tissue-specific modulation of apoptosis-related transcripts rather than uniform suppression of pro-apoptotic signaling. They may reflect the heterogeneous cellular composition of splenic tissue or differences in the timing of pathway-component responses, but those explanations were not tested.

The responses of apoptosis-related transcripts were tissue dependent. In the thymus, the HS group had higher *Caspase-3*, *Caspase-9*, *Cyt c*, and *Bak* mRNA abundance and lower *BCL-2* and *BCL-XL* mRNA abundance than the CON group. Dietary COS supplementation shifted most of these transcripts in the opposite direction, particularly at 400 mg/kg. This pattern is compatible with modulation of genes involved in the intrinsic mitochondrial apoptotic pathway, in which *Cyt c*, *Caspase-9*, *Caspase-3*, and *BCL-2*-family proteins participate [43,44,45]. Together with the thymic hemorrhage and cell necrosis observed histologically, the transcriptional changes are compatible with thymic injury in the HS treatment setting. However, they do not establish mitochondrial stabilization, caspase activation, or apoptosis because neither protein activation nor apoptotic cells were measured directly.

The bursa of Fabricius also showed a mixed transcriptional response: *Bak* was higher, but *Caspase-9* and *Cyt c* were lower in HS than CON, while *Caspase-3*, *BCL-2*, and *BCL-XL* did not differ. COS-associated bursal changes also varied by gene and dose. Across organs, the thymus showed the most internally coherent shift in apoptosis-related transcripts, whereas spleen and bursa responses were heterogeneous. Differences in lymphocyte maturation, cellular composition, and tissue microenvironment could contribute, but these possibilities were not tested. The stronger changes in *IFN-γ* and *IL-1β* mRNA at 200 mg/kg and the more consistent thymic apoptosis-related transcript pattern at 400 mg/kg suggest that inflammatory and cell-death-related readouts may respond over different dose ranges.

Several limitations constrain interpretation. The sampled tissue *n* was six per group. No thermoneutral + COS groups were included. Histology was qualitative, not blinded or morphometric, and did not include the bursa. Antioxidant assays were limited to spleen and thymus, and several assay-quality details require confirmation from original laboratory records. Most mechanistic endpoints were mRNA rather than proteins or functional immune measures. Finally, no global multiplicity correction or formal COS dose–response contrast was performed. Room replicated factorial studies with larger samples, thermoneutral COS controls, quantitative blinded histology, expanded redox measures, and protein and functional validation are needed.

Taken together, COS supplementation was associated with differences in immune-organ indices, serum corticosterone, measured oxidative status, and stress-, cytokine-, and apoptosis-related transcripts under cyclic HS. The response was organ and dose dependent: the thymus showed the most coherent apoptosis-related transcript pattern, while spleen and bursa responses were more heterogeneous. These data generate hypotheses for confirmatory studies rather than establish a single protective mechanism or an optimal dose.

## 5. Conclusions

In broilers under cyclic heat stress, dietary COS was associated with higher spleen and bursa indices, lower serum corticosterone, improved values for the measured splenic and thymic antioxidant/lipid-peroxidation endpoints, and tissue-dependent shifts in stress-, cytokine-, and apoptosis-related mRNA. The thymus showed the most coherent apoptosis-related transcript pattern, whereas spleen and bursa responses were heterogeneous. The 200 mg/kg group generally showed larger changes in pro-inflammatory transcripts, whereas 400 mg/kg showed the most consistent thymic apoptosis-related transcript pattern; no single dose was optimal across all outcomes. Because treatment was assigned at the room level, the CON versus HS contrast was confounded with room temperature condition. In addition, functional and protein-level validation was lacking. Therefore, these exploratory associations do not establish improved immune function and require validation in future room-replicated factorial studies.

## Figures and Tables

**Table 1:** Primers used for RT-qPCR.

Genes	Primer Sequence (5′→3′)	NCBI Sequence	Product Length (bp)
*β-actin*	F: CGTGACCTGACGGACTACCT R: TGCTCGAAATCCAGTGCGAC	NM_205518.2	128
*IL-1β*	F: CAAACTGCTGCGGAGGC R: AGGACTGTGAGCGGGTGTAG	NM_204524.2	149
*IFN-γ*	F: ACAGCCAACGCCAAAGC R: GGTGAAGGTTGCGAGGC	NM_205149.1	114
*IL-4*	F: AGCACTGCCACAAGAACC R: GCTAGTTGGTGGAAGAAGGT	NM_001007079.2	160
*IL-10*	F: GCTGAGGGTGAAGTTTGAG R: TGATGACTGGTGCTGGTCT	NM_001004414.4	192
*Hsp70*	F: CGGGCAAGTTTGACCTAA R: TTGGCTCCCACCCTATCTCT	NM_001167718.2	250
*Hsp90*	F: GTTGCGGAGAAGGTGACAGTGATC R: CTCCTTGATTCGCCGTTCTTCCAG	NM_001109785.2	186
*BCL-2*	F: CTTCCGTGATGGGGTCAACT R: AGGTACTCGGTCATCCAGGT	NM_205339.3	132
*Caspase-3*	F: CCATGGCGATGAAGGACTCT R: CCCGCTAGACTTCTGCACTT	NM_204725.2	99
*Caspase-9*	F: TCGTGGTCATCCTCTCCCAT R: CGATGTCTGACACCCGAAGT	XM_046931415.1	280
*Bak*	F: CTGTTCGCTTCCTTCCCCTG R: TTGCAGAGATGCTGTGGGAC	NM_001030920.3	166
*BCL-XL*	F: GCATCGTGGCTTTCTTCTCC R: CAGTACCCGCATCTCCTTGT	NM_001025304.1	71
*Cyt c*	F: TGGGCGCACTTACTGACAAT R: TGGAAGATGCCAAGACCCAC	NM_001398298.1	300

## Data Availability

The data presented in this study are available on request from the corresponding author.

## Supplementary Materials

The following supporting information can be downloaded at: https://www.mdpi.com/article/10.3390/ani16203244/s1, Animal welfare, Supplementary Table S1: Nutritional Level of Base Diet.

## Abbreviations

The following abbreviations are used in this manuscript:

HS	Heat stress
COS	Chitosan oligosaccharides
SOD	Superoxide dismutase
CAT	Catalase
GSH-Px	Glutathione peroxidase
MDA	Malondialdehyde
