# Supplementary material for: Dietary Chitosan Oligosaccharides Modulate Oxidative Status, Inflammatory and Apoptosis-Related Markers in Immune Organs of Heat-Stressed Broilers
Source: Animals (Basel). 2026 Oct 15;16(20):3244. doi: 10.3390/ani16203244 (PMC13700566; doi:10.3390/ani16203244)
Supplement: Supplementary file 1 [file animals-16-03244-s001.zip › Supplementary Table S1.pdf]

Supplementary Table S1. Nutritional Level of Base Diet (% of Air-Dried Basis)

Ingredients	1-42d	Calculated nutrient levels	1-42d
Corn	55.00	ME/(MJ·kg^{-1})	12.80
Soybean meal	34.84	Crude Protein	19.92
Wheat bran	2.00	Calcium	0.93
Soybean oil	5.00	Total Phosphorus	0.44
Shell flour	0.50	Methionine	1.30
Salt	0.30		
DL-Methionine	0.18		
L-Lysine	0.10		
Choline Chloride	0.10		
Vitamin Premix	0.20		
Mineral Premix	0.20		

Note: The premix provides vitamins per kilogram of feed: Vitamin A 9000 IU, Vitamin D3: 3240 IU, Vitamin E: 6 IU, Vitamin K3: 0.75 mg, Vitamin B1: 1.5 mg, Vitamin B2: 4.5 mg, Vitamin B6: 1.5 mg, Vitamin B12: 10 mg, Niacinamide: 10.5 mg, Folic Acid: 0.45 mg, Pantothenic Acid: 9 mg. 2) The premix provides the following mineral premix per kilogram of feed: Cu 5–10 mg, Fe 45–120 mg, Mn 45–85 mg, Zn 50–80 mg, Se 0.2 mg, I 0.15 mg. 3) Crude protein, total phosphorus, and calcium content are measured values; all others are calculated values. Refer to the "Agricultural Industry Standard of the People's Republic of China—Chicken Feeding Standards"(NY/T33-2004).
